# A Novel, Minimally-Invasive Approach to Repair Degenerative Disk Disease in an Ovine Model Using Injectable Polymethyl-Methacrylate and Bovine Collagen (PMMA/BC)

**DOI:** 10.7759/cureus.729

**Published:** 2016-08-08

**Authors:** Reid Hoshide, Erica Feldman, Anisha Narayan, William Taylor

**Affiliations:** 1 Department of Neurosurgery, University of California, San Diego

**Keywords:** minimally invasive spine surgery, degenerative disk disease, neurosurgery, orthopedic surgery

## Abstract

Background :

The natural, inflammatory repair processes of an injured intervertebral degenerative disc can propagate further injury and destruction. While there are many different treatment modalities of the pain related to degenerative disc disease, none are actually reparative in nature. Treatment strategies to repair a degenerative disc without inducing a destructive inflammatory milieu have been elusive.

Purpose:

The purpose of this experiment is to discover the feasibility of reconstructing an injured intervertebral disc using an injected, inert polymer as the foundation for endogenous collagen growth.

Study Design:

In this ovine model of six subjects in total, we introduce a modality where a large inert polymer, polymethyl methacrylate (PMMA), in conjunction bovine collagen (BC) is injected into the intervertebral disc. Following six months of observation, histologic specimens were evaluated macroscopically and microscopically for evidence of a benefit of the injectable PMMA/BC.

Methods:

We obtained six merino sheep for this study. Concentric injuries were made to four of their lumbar intervertebral discs. Two of those levels were treated with a percutaneous injection of 0.3 cc of PMMA/BC. The remaining lumbar levels were left untreated and were our controls. After six months, all subjects were sacrificed. Their four levels were extracted and were examined macroscopically and microscopically.

Results:

All subjects tolerated the lumbar injury and percutaneous injection of PMMA/BC well. After the six month interval, all subjects have demonstrated an intact architecture of their lumbar disc height at the macroscopic and microscopic level. Microscopically, there was no evidence of external migration of the PMMA/BC microspheres, nor was there any evidence of an inflammatory response by its presence. Notably, the PMMA/BC microspheres were well-incorporated into the concentric disc tears and had undergone endogenous collagen formation in its environment. Treatment levels were revealing for maintenance of disc height without evidence of an ongoing degeneration. The controlled levels were revealing for continued disc degeneration with loss of disc height and evolving injury at the level of the concentric tear.

Conclusions:

This ovine model demonstrates a novel and promising technique for prevention and arrest of lumbar intervertebral disc degeneration.

## Introduction

Lower back pain is an extremely common condition, affecting as many as 80% of adults at some point in their lives [1-2]. The prevalence of lower back pain symptoms, which are reported to have doubled in the past 40 years [3], afflict populations worldwide [4-6]. Lower back pain is one of the most widespread reasons people seek medical care. It is also one of the most common ailments that decrease worker productivity, and has a significant universal impact on families, communities, and economies [2, 4, 7]. A frequent cause of lower back pain is degenerative disc disease (DDD), a chronic condition characterized by progressive intervertebral disc injury [8-9]. While DDD can be asymptomatic, its diagnosis is often attributed to a wide array of symptoms including axial skeletal pain, radiculopathy, and myelopathy. Degenerative disc disease can occur naturally during the process of senescence, as a result of decreased vascular supply and nutrient flow to the discs and adverse changes in cellular activity [10-11]. Degenerative disc disease has also been observed in younger adults who experience back trauma or excessive spinal movements, causing tears within the disc border which accelerate the degenerative processes [12]. As degeneration progresses, the intervertebral disc desiccates, and therefore loses its ability to absorb physiological loads efficiently. This strain causes loads to redistribute to the adjacent vertebral bodies, resulting in endplate changes, osteophyte production and trabecular microfractures [13].

**Figure 1:**
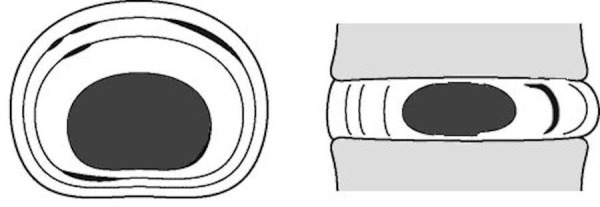
Illustration of a concentric annular injury, where the normal scalloping of the annulus fibrosus is expanded between the concentric lamellar architecture.

A healthy intervertebral disc has four distinct tissue types: the annulus fibrosus, the transition zone, the nucleus pulposus, and the endplate [14-15]. The annulus fibrosus, a strong material that makes up the perimeter of the disc, is composed primarily of type I collagen which exhibits robust tensile strength. The outer portion of the annulus fibrosus is composed of about 30-90 layers of collagen fibrils called lamellae. A less-dense inner portion of the annulus fibrosus contains fibroblast-like cells and lacks organization. The annulus is often the first site to be injured in the onset of DDD, after which scar tissue resulting from the healing of small tears can cause progressive weakening of the disc [12]. An injured annulus is represented by a concentric annular tear, where the normal scalloping of the layers of the annulus fibrosus is expanded by an injury. Figure 1 illustrates this type of injury. 

The transition zone is a thin fibrous tissue separating the annulus from the nucleus pulposus. The nucleus pulposus is the gelatinous core composed of chondrocyte-like cells and notochordal cells. The nucleus is composed of mainly type II collagen, which exhibits less organization and strength when compared to type I collagen. The endplate provides superior and inferior containment through a thick layer of articular cartilage and is the conduit for the vascular supply that maintains and upkeeps the intervertebral disc elements.

Pathophysiologic models of DDD includes upregulation of proinflammatory cytokines, which increases secretion of matrix metalloproteinases, thereby inducing the breakdown of the extracellular matrix via enzymatic degradation [16]. Major downstream biochemical changes resulting from DDD include: decreased cell density, loss of proteoglycan, loss of hydration due to decreased osmotic pressure, alteration of collagen architecture, endplate sclerosis, and altered biomechanics which can eventually cause failure of disc function and result in pain and deformity of the spine [16]. The induction of the body’s natural repair processes for these defects often results in ingrowth of the nerve or blood vessels beyond their normal location in the outer third of the annulus. Since the inflammation caused by this repair mechanism plays an integral role in disc function failure, effective treatment options for DDD would ideally involve the ability to facilitate this reparative process without the induction of an inflammatory response.

Current first-tier management for patients with symptomatic DDD involves non-invasive measures such as rest, oral anti-inflammatory medications, and physical therapy. Second tier management is somewhat more invasive and includes corticosteroid injections into the epidural space or thermal and radiofrequency ablation into the disc space. These more invasive treatment strategies are often distributive throughout the disc space. Third and final tier interventions include costly and highly invasive surgical procedures such as discectomy, laminectomy, fusion, or total disc replacement.

This current study presents a plausible solution involving the use of a microsphere collagen bulking agent, polymethyl-methacrylate and bovine collagen implant (PMMA/BC), to promote endogenous healing of an injured intervertebral disc. PMMA is an inert polymer with smooth spherical surfaces [17]. These properties allow the body’s endogenous collagen to encapsulate the PMMA microspheres and thus add structure to the environment once it is implanted [17]. PMMA/BC microspheres are 30-50 µm in diameter, an optimal size to escape phagocytic action by macrophages and giant cells [18]. Also, PMMA/BC microspheres are not susceptible to enzymatic activity, and can promote greater collagen deposition by presenting a larger surface area for ease of injectability [18]. The function of the microspheres is to provide a scaffold to promote constant deposition of newly synthesized endogenous collagen. Its success has been proven as a cosmetic, dermatologic filler in conditions that require structural bulking [17, 19]. A percutaneous treatment using PMMA/BC microspheres is used in this study to enhance the reparative process of a concentrically-injured annulus fibrosus to maintain normal disc cytoarchitecture. The PMMA/BC microspheres can be implanted into the disc space via direct injection of a concentric annular tear with fluoroscopic assistance or by locally injecting the exposed annulus through a surgical corridor. In choosing this agent, we hypothesize that the natural reparative process in the annulus can be enhanced by sealing off annular tears, which would ideally allow for the maintenance of normal disc cytoarchitecture. Moreover, the lack of an inflammatory response to PMMA/BC’s presence creates an environment of healing that lacks collateral injury that would otherwise occur with the inflammatory process. The ability to intervene using a reparative process in degenerative disc disease that maintains disc integrity, as opposed to a more destructive process, is the basis for the proposed treatment approach.

We seek to determine if PMMA/BC would be a viable modality to add to current tier II options to improve structural stability of the injured intervertebral disc thereby obviating the need to graduate to more invasive interventions.

## Materials and methods

An ovine animal model was utilized due to its biomechanical similarity to the lumbar intervertebral discs in humans [20]. This study was approved by our Institutional Animal Care and Use Committee (IACUC). The methodology employed by Osti et al. [20] to morphologically classify and produce annular defects was implemented for the study. The study model introduced concentric annular tears within the lumbar discs of the ovine model. Four-disc levels of each of six total middle-aged merino wethers sheep were used for the study. 

After surgical exposure of the lumbar discs using a left retroperitoneal approach, a concentric injury was made via blunt traction on the annulus of the left outer quadrant of the disc. The injury was made uniformly at four contiguous lumbar levels (two of which will serve as the "treatment" levels, and the other two levels as untreated controls). The surgical incision was then sutured closed. Immediately following this, the treatment levels were injected with 0.3 cc of PMMA/BC solution (20% PMMA, 3.5% bovine collagen, suspended in buffered, isotonic water) at the inner annular nuclear junction using fluoroscopic guidance, which guided us exactly to the injured areas based on the measurements made during the injurious process. The assignment of treatment and control levels were randomized. 

The subjects then emerged from the perioperative anesthesia and were allowed to live without restriction for six months within an observed environment. An independent veterinarian performed daily assessments to ensure the safety and well-being of each subject. Specifically, the subjects were also observed for any signs and symptoms of neurologic compromise related to the procedure. 

After six months, the lumbar spines were extracted for review. Each lumbar spine was trimmed into its functional spinal components, fixated in 10% neutral buffered formalin, decalcified, and cut transversely through the intervertebral space to yield histologic sections of the cranial and caudal regions for macroscopic and microscopic analysis. Axial and coronal slices were prepared using hematoxylin and eosin (H&E) for microscopic analysis.

The histological samples were evaluated by an independent pathologist for the purpose of detecting the following physiological responses: degenerative changes of control and PMMA/BC groups, PMMA/BC incorporation, type and extent of reparative processes, degree of inflammatory and immunogenic responses, new collagen formation, and migration and ingrowth of nerve or blood vessels.

## Results

Pre- and post-operatively, each subject was examined by a veterinary neurologist, and no clinical neurologic decline or deficit was noted. The subjects had no activity or meal restrictions during the observational period. A single subject died three weeks post-operatively from an isolated, unrelated pulmonary infection as determined by necropsy. This subject’s lumbar spine was extracted for review following the necropsy and represented a 3-week evaluation. After six months of observation, the remaining subjects were sacrificed. Microscopic and macroscopic histopathologic specimens from the lumbar vertebral sites and several planes were reviewed and reported.

**Figure 2:**
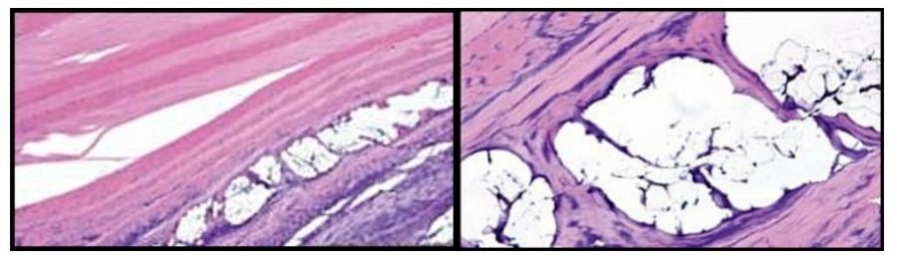
Three weeks post PMMA/BC implantation (Left: 10x magnification, H&E stain), (Right: 40x magnification, H&E stain). *Histologic H&E sections revealed maintenance of the PMMA/BC microspheres without migration beyond the lamellar injury. *Healing of the annular defect occurred with early signs of the formation of new collagen without an inflammatory response. Most of the microspheres were lost during histologic preparation, and blue material observed within the voids is likely proteinaceous fluids or retained collagen from the injected PMMA/BC. There is a minor amount of fibrous tissue smoothing the internal layer of the voids.

At the three-week mark, from the early-deceased subject, it was noted that the PMMA/BC microspheres were well incorporated within the annular lamellae, as demonstrated through microscopic histologic examination seen in Figure 2. 

Additionally, small amounts of collagen formation can be seen within the microsphere environment. This appears to be an early, organized formation of collagen, thus unlikely to be the implanted bovine collagen as part of the PMMA/BC solution. No inflammatory cells were seen. 

**Figure 3:**
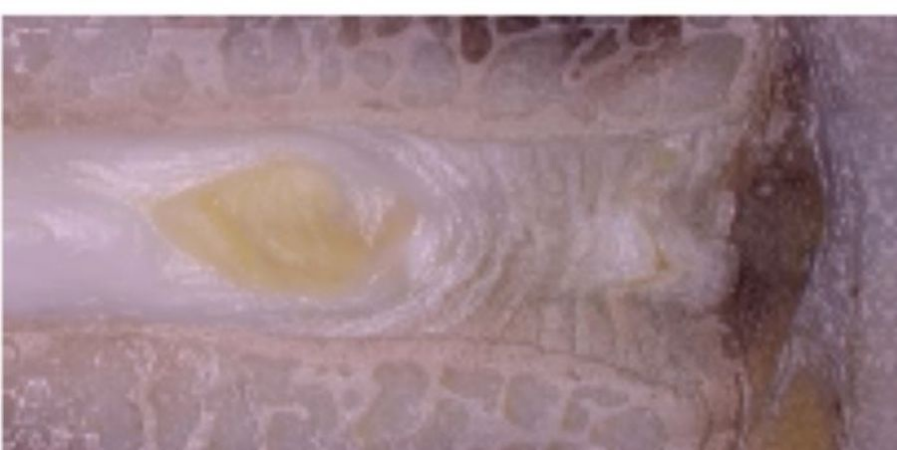
Gross specimen of our treatment level, showing the area of injury and PMMA/BC injection after six months. There is an apparent prevention of disc degeneration and with the maintenance of disc height and integrity.

At the six month mark, gross specimens were reviewed. Figure 3 represents a level that was treated by the PMMA/BC, showing evidence of healing at the area of injury.

**Figure 4:**
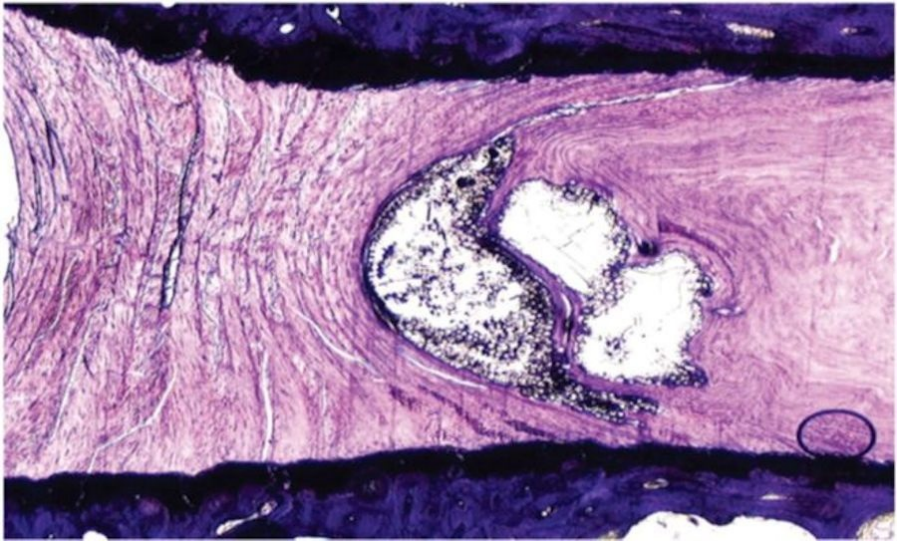
H&E slide showing prevention of disc degeneration in our treatment specimen. Prevention of disc degeneration is indicated by lack of disc narrowing, radial bulging, and vertebral osteophyte formation. Additionally, no nuclear clumping or failure of annular patency led to disc space collapse and annular scarring. Despite inflicted mechanical injury, the annulus shows normal architecture without showing progressive degenerative changes.

Moreover, the figure demonstrates maintenance of disc height, macroscopic lack of inflammation, and lack of vertebral osteophyte formation. Following formalin fixation and histologic preparation, the intervertebral disc has incorporated the PMMA/BC microspheres within the annular defect seen in Figure 4.

**Figure 5:**
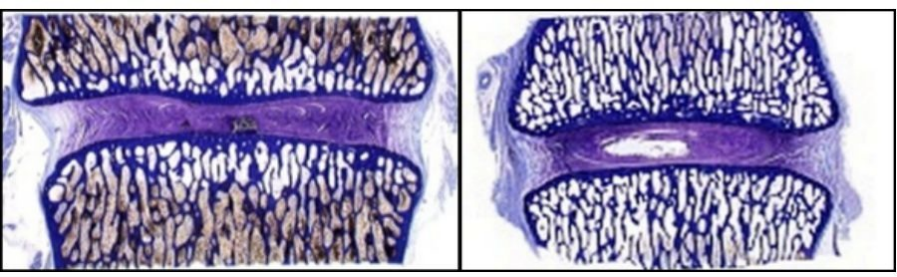
Image illustrating collapse of disc height and vertebral osteophyte formation (left) compared to treatment group whose disc height has been maintained (right).

Both untreated injury (control) specimens showed progressive degenerative changes which worsened over six month period when compared to the treatment specimens as shown in Figure 5. 

**Figure 6:**
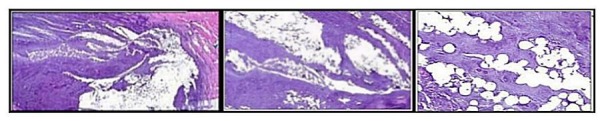
H&E slides show lack of any apparent inflammatory response seen in the annulus after 6 months. (Left: 1.25x magnification, Middle: 4x magnification, and Right: 20x magnification) . Large amounts of microspheres found within the annular lamellae with new collagen formation. Microspheres are present within tissue overlying annulus with an evident fibrotic response.

These degenerative changes were seen macroscopically as disc narrowing, radial bulging, and vertebral osteophyte formation, along with nuclear clumping. Additionally, failure of annular patency led to disc space collapse. Microscopically, the H&E stains did not reveal any migration of the PMMA/BC microspheres from the inner and the outer annular fibers. This is demonstrated in Figure 6.

The injection of PMMA/BC lacked any local scarring seen at the annular/nuclear border. Importantly, no abnormal tissue response including inflammatory tissue or angiogenesis was seen in the treated discs. There was no macroscopic or microscopic evidence of microsphere extravasation within or outside the disc at any level in any specimen. The injection in the annulus did result in new collagen formation and was not accompanied by inflammatory changes.

## Discussion

Results from this animal model on the use of PMMA/BC, an injectable bulking agent for prevention of degenerative disc disease progression, led to novel findings. First, the study confirmed that endogenous collagen growth can be induced by injection of PMMA/BC in areas that have minimal but available blood supply including the outer to mid annulus. This shows promise for natural, endogenous collagen repair of annular tears outside of the inner annulus in humans. The lack of neovascularity or nerve ingrowth along with the lack of inflammatory cells reveals a possible opportunity for normal repair process after injection with PMMA/BC, which may be beneficial in preventing and repairing degenerative changes. This possibility may be the topic of future studies.

The PMMA/BC-treated disc showed maintenance of normal architecture and an arrest of degenerative changes when compared to the placebo levels where continued disc degeneration was demonstrated. The treatment levels revealed disc height maintenance with the lack of appreciable microsphere migration or inflammatory response. 

Based on the results observed in other injection sites [21-23], the results of our study were similar and expected results. In each location where the microspheres were exposed to a blood supply, a new collagen formation occurred at the site of injected PMMA/BC without the presence of an inflammatory response.

## Conclusions

We have demonstrated that annular healing occurred in the treatment levels in our ovine model with annular augmentation of PMMA/BC to sites of injury. We conclude that PMMA/BC is a novel and promising product that has future potential for treating degenerative disc disease with a biochemical, tier II approach.

​
